# The Role Played by Imidazole Propionic Acid in Modulating Gut-Heart Axis and the Development of Atherosclerosis: An Explorative Review

**DOI:** 10.7759/cureus.94362

**Published:** 2025-10-11

**Authors:** Venkata BharatKumar Pinnelli, Jayashankar CA, Venkataramana Kandi, Spandana P, Manish GR, Mir Hyder Hussain, Akshay AS, Kavitha R, Ramya JP, Surendra Babu T, Sabitha Vadakedath

**Affiliations:** 1 Biochemistry, Vydehi Institute of Medical Sciences and Research Centre, Bangalore, IND; 2 Internal Medicine, Vydehi Institute of Medical Sciences and Research Centre, Bangalore, IND; 3 Clinical Microbiology, Prathima Institute of Medical Sciences, Karimnagar, IND; 4 General Medicine, Vydehi Institute of Medical Sciences and Research Centre, Bangalore, IND; 5 Anatomy, Vydehi Institute of Medical Sciences and Research Centre, Bangalore, IND; 6 Biochemistry, Prathima Institute of Medical Sciences, Karimnagar, IND

**Keywords:** atherosclerosis, biomarker, cardiovascular disease, coronary artery disease, dysbiosis, gut-heart axis, gut microbiome, gut microbiota, imidazole propionic acid, therapeutic target

## Abstract

Recent studies have demonstrated the significant role of the normal human microbial component, also known as gut microbiome/gut microbiota (GM). Dysbiosis, or imbalance of GM, can predispose to systemic diseases, including cardiovascular disease (CVD). The GMs' influence extends further to cardiometabolic health, with microbial metabolites playing a pivotal role in these interactions. Traditional risk factors like hyperlipidemia and hypertension are now complemented by emerging evidence implicating GM-derived metabolites in the pathogenesis of atherosclerosis (ATS). Imidazole propionic acid (ImP), a metabolite of histidine derived from GM, has emerged as a significant mediator linking GM dysbiosis to ATS and CVD, or coronary artery disease (CAD). This comprehensive review synthesizes current knowledge on ImP’s biosynthesis, molecular mechanisms, clinical relevance, and therapeutic potential, emphasizing its role in the gut-heart axis and cardiovascular pathology. Appropriate keywords, including "microbes", "dysbiosis", "gut microbiota/gut microbiome and cardiovascular disorders", "atherosclerosis and microbes", and "microbial metabolites", among others, were used to extract relevant studies in PubMed and Google Scholar from inception to date. ImP bridges microbial dysbiosis and CVD through endothelial dysfunction, inflammation, and metabolic disturbances. Its production is modifiable by diet and GM composition, positioning ImP as both a biomarker and therapeutic target in ATS and heart failure. Advancing understanding of ImP’s biology and clinical impact will enable novel interventions to reduce the global burden of atherosclerotic cardiovascular disease (ASCVD), marking a change in basic assumptions in cardiovascular medicine centered on the gut-heart axis.

## Introduction and background

The human gut microbiota/gut microbiome (GM) is a complex and dynamic collection of microorganisms that are essential for human health and survival. GM is formed shortly after birth and persists throughout a person's life. These bacterial communities found in numerous physiological systems, such as the skin, gastrointestinal tract (GIT), urogenital tract, and respiratory tract, are known as normal human microbial flora. It has been described as diverse and stable, with a shared core microbiota, and as an important regulator of body homeostasis, influencing a wide range of physiological activities such as metabolism, homeostasis, inflammation, and hemopoiesis. Any alteration in the composition of this community, referred to as dysbiosis, causes not only GIT difficulties but also affects other organs and might result in interconnected illnesses [1-5]. GM has been described as an essential element that interacts with other organs and creates a reciprocal connection or axis between the organs, including the gut-skin, gut-liver, gut-kidney, gut-brain, and gut-heart [4]. GM dysbiosis has been linked to greater vulnerability to respiratory illnesses, as well as changes in immune response and lung homeostasis. Several studies have reported the existence of GM dysbiosis during coronavirus disease 2019 (COVID-19) and its correlation with illness severity [6, 7]. Recent developments demonstrate the potential of beneficial microbes to treat diseases and improve health, in addition to offering potentially promising alternatives to traditional medications. Numerous ways that bacteria improve health have been made clear by the surge of investigation into GM. Furthermore, novel approaches, like treating complicated and chronic human diseases with engineered living microorganisms (ELMs), have recently gained significance. The positive uses of microbes, such as prebiotics, probiotics, and postbiotics, have been widely recognized. Prebiotics are food for bacteria, generally include high-fiber foods that can be utilized by microorganisms to promote the growth of beneficial bacteria in the gut, even though the body cannot digest them. Foods containing living bacteria called probiotics are meant to enhance or repair the GM. Yoghurt, cheese, various fermented foods, and therapeutic capsules with ELMs are examples of probiotics. Beneficial, non-viable elements known as postbiotics include metabolites, inactivated microbial cell components that benefit the host's health. They work by keeping pathogens from overgrowing and reestablishing the natural human flora. Additionally, bacteria have been employed in fecal microbiota transplantation (FMT) to treat irritable bowel disease (IBD) patients and antibiotic-associated diarrhea caused by *Clostridium difficile *[8-12]. Bacteria contribute to regenerative medicine (RM) applications by modifying the immune response, producing helpful biomolecular compounds, and controlling the regenerating/healing process. Bacteria also produce other metabolites that can be used in tissue engineering to scaffold and transport drugs. The general composition of the GM can either impede or facilitate processes such as wound healing and GIT restoration, making microbial modification a possible therapeutic tool [13-15].

**Figure 1:**
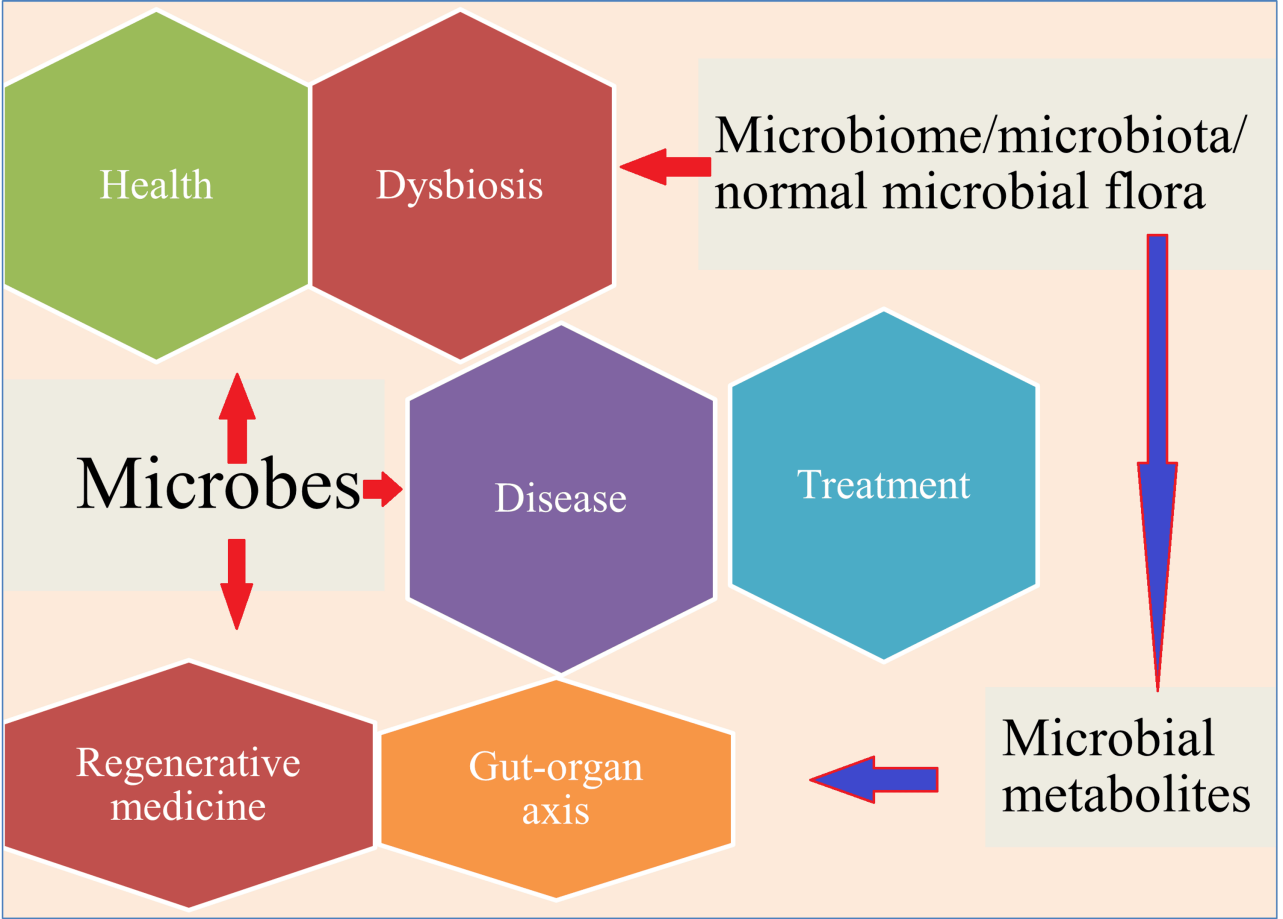
The influence of microbes on health, disease, and treatment This image has been synthesized from references [1-21].

The effective roles of microbial metabolites have been discovered as a result of developments in the disciplines of metabolomics and microbiome analysis. These discoveries have demonstrated significant potential for identifying new treatment targets for human illnesses. Even with these developments, there is still much to learn about unknown microbial metabolites and their effect on health and illness [16]. Some of the microbial metabolites implicated in human health and diseases include aromatic amino acid, reduced glutathione (GSH), oxidized glutathione (GSSG), 3-(3-hydroxyphenyl)-3-hydroxypropionic acid (HPHPA), S-adenosyl-L-homocysteine (SAH), S-Adenosyl-L-methionine (SAM), short-chain fatty acid (SCFA), trimethylamine N-oxide (TMAO) [17]. The GM's makeup has become a crucial regulator of tumor growth, modulating both innate and adaptive immunity. Uncertainty surrounds the fundamental mechanisms by which the GM influences tumor formation. Antitumor immunity is fueled by the GM-derived metabolite TMAO, which also establishes the foundation for treatment approaches [18]. A recent study found that the breakdown of bile acid metabolites and tryptophan generated from diet aided by GM promotes brain cell development and function while preventing inflammation of the central nervous system (CNS) [19]. Globally, cardiometabolic disorders are now a major source of illness and mortality. The taxonomical and functional composition of the GM has been strongly associated with them, and some of the connections may be mediated by food/nutrition. The ability to modify GM and alter diet creates opportunities for new treatment approaches [20]. GM's metabolic activities have been related to increased risk for heart failure (HF), atherosclerosis (ATS), and serious cardiovascular (CV) events like stroke and myocardial infarction (MI). The immune system's reaction to an injury or foreign material is inflammation, which is one way that an unhealthy GM compromises health. Substances that leave the gut and enter the bloodstream, such as compounds formed by GM, participate in gut inflammation. Therefore, dysbiosis and pathogen invasion trigger an inflammatory response. Blood arteries that are affected by inflammation lose their flexibility, the function of the endothelium wall is compromised, and the ensuing pathophysiological conditions result in ATS (Figure 1) [21].

The growing body of evidence supporting imidazole propionic acid (ImP), a GM-derived metabolite, and its pathogenic role in ATS represents a change in thinking in our understanding of cardiovascular disease (CVD) etiology, emphasizing the importance of the GM as a modifiable risk factor and potential therapeutic target in CV medicine [22]. This review aims to provide a comprehensive analysis of current knowledge regarding ImP and its role in ATS, examining its biosynthetic pathways, molecular mechanisms of action, clinical associations, and therapeutic potential. By synthesizing findings from epidemiological studies, mechanistic investigations, and experimental models, this review aims to establish ImP as a critical mediator in the gut-heart axis and to highlight future research directions that may lead to improved CV outcomes. 

## Review

Imidazole propionic acid and atherosclerosis

ATS remains a leading cause of CV morbidity and mortality worldwide, characterized by the progressive accumulation of lipids, inflammatory cells, and fibrous elements within arterial walls. While traditional risk factors such as hyperlipidemia, hypertension, and diabetes mellitus (DM) have been extensively studied, emerging evidence highlights the critical role of GM-derived metabolites in CVD pathogenesis [23, 24]. Among these bioactive compounds, ImP, a histidine-derived metabolite produced by specific bacterial species, has garnered significant attention as a novel mediator of atherosclerotic cardiovascular disease (ASCVD). ImP is synthesized through the microbial metabolism of L-histidine via the urocanate reductase (UrdA) pathway, primarily by bacterial species including *Ruminococcus gnavus *and *Veillonella *[25]. This metabolite represents a paradigmatic example of the gut-heart axis, demonstrating how GM can influence CV health through the production of bioactive compounds that enter systemic circulation and exert distant effects on vascular tissues [23, 24].

Recent clinical investigations have established compelling associations between elevated plasma ImP levels and CVD risk. In a comprehensive study of 831 patients undergoing cardiac angiography, ImP concentrations were significantly higher in individuals with coronary artery disease (CAD), with those in the highest quartile demonstrating a four-fold increased risk of CAD even after adjustment for traditional CV risk factors [23]. These findings have been corroborated across diverse populations, including individuals with Human Immunodeficiency Virus (HIV) infection, where ImP levels correlate with obstructive CAD severity [25]. The pathophysiological significance of ImP extends beyond simple disease association. Mechanistic studies have revealed that ImP directly impairs endothelial cell function through disruption of the insulin receptor signaling cascade, specifically targeting the phosphatidylinositol 3-kinase (PI3K)/protein kinase B (PKB) pathway and leading to sustained activation of the forkhead box protein O1 (FOXO1) transcription factor [22, 26]. A vital intracellular signaling system, PI3K/PKB, plays a role in angiogenesis, metabolism, growth, survival, and proliferation of cells. FOX01 is implicated in diseases, including DM, cancer, and metabolic conditions. It also controls several biological functions, such as the cell cycle, death, deoxyribonucleic acid (DNA) repair, and metabolism. Molecular disruptions in PI3K/PKB and FOXO1 result in the progression of ATS, attributed to compromised endothelial migration, reduced angiogenic capacity, and enhanced inflammatory responses [23].

**Figure 2:**
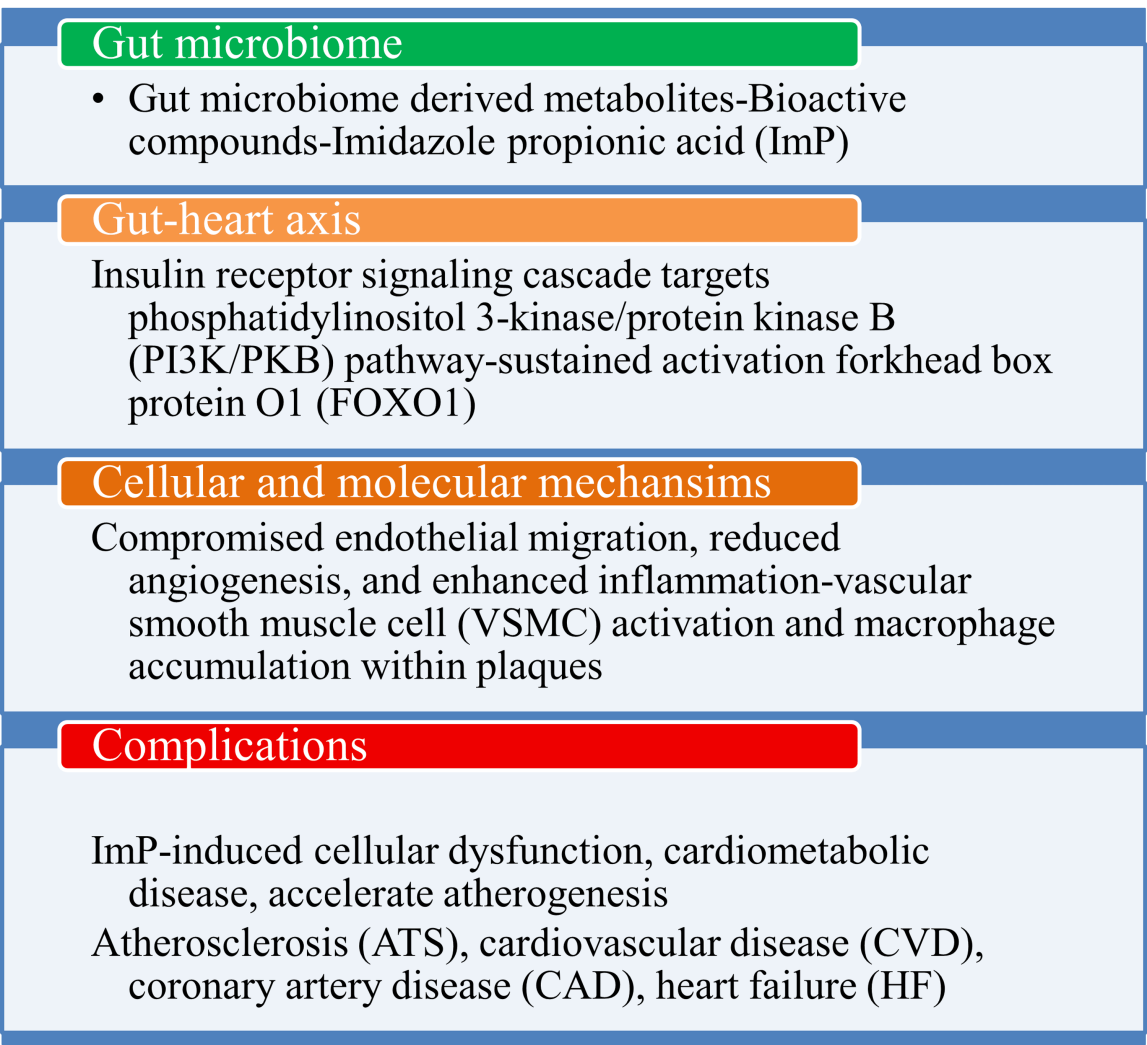
Probable mechanisms behind imidazole propionic acid induced cardiac complications This image has been synthesized from references [23-26]. PI3K/PKB: phosphatidylinositol 3-kinase/protein kinase B; FOX01: forkhead box protein O1; VSMC: vascular smooth muscle cell; CVD: cardiovascular disease; CAD: coronary artery disease; HF: heart failure; ATS: atherosclerosis

Animal model investigations have provided crucial evidence for ImP's causal role in ATS development. In ATS-prone apolipoprotein E (ApoE) deprived mice, chronic ImP administration significantly increased ATS independent of cholesterol levels, accompanied by enhanced vascular smooth muscle cell (VSMC) activation and macrophage accumulation within plaques [23]. These findings demonstrate that ImP can directly accelerate atherogenesis through lipid-independent mechanisms. The clinical relevance of ImP extends to HF, where elevated levels predict overall survival and correlate inversely with left ventricular ejection fraction [26]. Additionally, ImP has been implicated in DM-related complications, including diabetic nephropathy and impaired wound healing, suggesting its role as a multifaceted mediator of cardiometabolic disease [24]. From a therapeutic perspective, ImP represents an attractive target for CVD prevention and treatment. ImP's production can potentially be modulated through dietary interventions, probiotic supplementation, or direct enzymatic inhibition of the UrdA pathway [22, 23]. Recent studies have identified compounds such as pirfenidone that may counteract ImP-induced cellular dysfunction, opening new avenues for therapeutic development (Figure 2) [24].

Pathophysiology of atherosclerosis

ATS represents a chronic inflammatory disease of large and medium-sized arteries, characterized by the progressive accumulation of lipids, inflammatory cells, fibrous elements, and calcification within the arterial wall [27]. This multifactorial disease process involves the formation of fibrofatty lesions, termed atherosclerotic plaques or atheroma, which develop through a complex cascade of cellular and molecular events that can span decades [28, 29]. The pathogenesis of ATS begins with endothelial dysfunction, representing the initial and critical step in atherogenesis [28, 29]. Endothelial activation occurs in response to various stimuli, including oxidized low-density lipoproteins (LDL), hemodynamic stress, inflammatory mediators, and infectious agents [30, 31]. This dysfunction manifests as increased vascular permeability, enhanced expression of adhesion molecules such as Intercellular adhesion molecule 1 (ICAM-1) and vascular cell adhesion molecule 1 (VCAM-1), and reduced nitric oxide (NO) bioavailability [32]. Following endothelial activation, the atherosclerotic process proceeds through several well-characterized stages. Lipid accumulation occurs as LDL particles penetrate the compromised endothelial barrier and become trapped within the subendothelial space, where they undergo oxidative modification [29, 32]. These oxidized LDL particles are recognized as foreign antigens, triggering an inflammatory response that attracts circulating monocytes to the arterial wall [33].

Inflammatory cell recruitment represents a hallmark of atherosclerotic progression [31, 32]. Monocytes adhere to activated endothelial cells via selectins and integrins, subsequently transmigrating into the intimal layer where they differentiate into macrophages [33]. These macrophages attempt to clear the accumulated lipids through phagocytosis but become overwhelmed by the excessive oxidized LDL, transforming into cholesterol-laden foam cells [32]. Foam cells release proinflammatory cytokines and chemokines, perpetuating the inflammatory cascade and recruiting additional immune cells, including thymus (T)-lymphocytes, dendritic cells, and neutrophils [32]. As the lesion progresses, VSMC migration and proliferation occur from the medial layer into the intima [28, 29]. VSMCs contribute to plaque stability through extracellular matrix synthesis, particularly collagen production that forms the protective fibrous cap [29]. However, VSMCs can also transform into foam cells upon lipid uptake, further contributing to plaque growth [32]. Advanced plaque development is characterized by the formation of a necrotic core containing apoptotic and necrotic cellular debris, cholesterol crystals, and extracellular lipids [29, 32]. The balance between apoptosis and efferocytosis (clearance of apoptotic cells) becomes disrupted in advanced ATS, leading to defective clearance mechanisms and secondary necrosis [29]. This process releases damage-associated molecular patterns (DAMPs) that amplify inflammation and promote further tissue damage [32].

Clinical significance of atherosclerosis

ATS serves as the fundamental pathological process underlying the majority of CVDs, representing the leading cause of morbidity and mortality worldwide [27, 34]. The clinical significance of ATS extends across multiple vascular territories, each associated with distinct but overlapping manifestations. CAD represents the most prevalent and deadly manifestation of ATS [34, 35]. Atherosclerotic plaques in the coronary circulation can cause chronic ischemia through progressive luminal narrowing or acute coronary syndromes following plaque rupture and thrombosis [35]. The latter mechanism accounts for the majority of MIs, where vulnerable plaques with thin fibrous caps and large necrotic cores are prone to rupture, exposing thrombogenic material to the circulation [28, 29] Cerebrovascular disease constitutes another major clinical consequence of ATS, particularly ischemic stroke [34, 35] Atherosclerotic plaques in carotid, vertebral, and intracranial arteries can cause stroke through similar mechanisms as CAD: either gradual stenosis leading to hemodynamic compromise or acute thromboembolism following plaque rupture [36]. Peripheral artery disease (PAD) affects the extremities, most commonly the lower limbs, causing claudication, rest pain, and in severe cases, critical limb ischemia [34]. The atherosclerotic process in peripheral arteries follows similar pathophysiological mechanisms but often presents with more indolent symptoms compared to CV or cerebrovascular manifestations [37]. Renal artery stenosis represents another clinically significant manifestation, potentially leading to renovascular hypertension and chronic kidney disease (CKD) [38]. The kidneys' high blood flow requirements make them particularly susceptible to atherosclerotic compromise [39].

Global burden and epidemiological impact of atherosclerosis

The epidemiological burden of ASCVD represents one of the most significant public health challenges of the 21^st^ century [34, 37]. Current estimates indicate that CVDs affect over 523 million people globally, with atherosclerotic diseases serving as the primary mediators of this burden [34]. Mortality trends reveal complex patterns across different regions and time periods [34, 35]. While high-income countries have experienced dramatic declines in ASCVD mortality since the mid-20th century-with some countries showing reductions from 22% to 6% in middle-aged men between 1950 and 2010-recent data suggest an attenuation of these favorable trends [32]. The Global Burden of Disease Study 2019 demonstrated that from 1990-2019, global prevalence rates of ischemic heart disease (IHD), ischemic stroke (IS), and PAD in young adults (20-54 years) increased substantially [37]. Age-specific patterns reveal concerning trends in younger populations [37]. The prevalence of IHD in the 20-54 age group increased by 20.55%, IS by 11.50%, and PAD by 7.38% over the three-decade study period [34]. These patterns contrast sharply with declining prevalence rates in older populations, suggesting evolving risk factor profiles and potential inadequacies in primary prevention strategies [37]. Socioeconomic disparities significantly influence ASCVD burden [34, 37]. Lower socio-demographic index (SDI) regions exhibit disproportionately higher rates of young ASCVD, with low SDI regions showing prevalence rates of 20.70% for IHD, 40.05% for IS, and 19.31% for PAD among young adults-higher than corresponding rates in high SDI regions [37]. Regional variations highlight the heterogeneous nature of the ATS epidemic [34, 35]. While most low- and middle-income countries (LMICs) have reported declines in stroke mortality, trends for IHD vary, with some Eastern European and Asian countries experiencing an increase rather than a decrease [35]. These disparities reflect differences in healthcare infrastructure, risk factor management, and socioeconomic development [37].

Atherosclerosis risk factor profile and disease progression

The multifactorial nature of ATS involves complex interactions between traditional risk factors, including dyslipidemia, hypertension, DM, smoking, and obesity [27, 36]. Recent evidence has expanded this framework to include emerging risk factors such as chronic inflammation, clonal hematopoiesis, and environmental exposures [27, 32]. Metabolic risk factors represent primary contributors to ASCVD burden across all age groups [37]. The Global Burden of Disease analysis identified inadequate control of high body mass index (BMI) and elevated fasting blood glucose (FBG) as particularly problematic in younger populations, potentially explaining divergent trends between age groups [37]. Environmental factors, particularly ambient particulate matter pollution, show increased impact on younger individuals compared to older adults [37]. The inflammatory paradigm has revolutionized the understanding of ATS pathophysiology [31, 32]. Recognition that ATS represents a chronic inflammatory disease at every stage, from initiation through progression to clinical complications, provides mechanistic frameworks for understanding how diverse risk factors converge on common pathways [31, 33]. This understanding has opened new therapeutic avenues and highlighted the importance of addressing systemic inflammatory burden in CV risk management [32]. Plaque vulnerability rather than simply stenosis severity determines clinical outcomes [29, 32]. Vulnerable plaques characterized by large necrotic cores, thin fibrous caps, and active inflammation are more prone to rupture and thrombosis than heavily calcified, fibrotic lesions [28, 29]. This knowledge has shifted focus from purely anatomical assessments toward comprehensive evaluation of plaque composition and inflammatory activity [32]. The evolving understanding of ATS as a systemic inflammatory disease with complex metabolic underpinnings provides the conceptual framework for investigating novel mediators such as GM-derived metabolites, including ImP, in CV pathogenesis [27, 31]. This background establishes the rationale for exploring how microbial metabolites may influence traditional atherosclerotic pathways and contribute to residual CV risk despite optimal management of conventional risk factors.

Gut microbiome

The human GM represents one of the most complex and metabolically diverse ecosystems within the human body, comprising 1,000-1,150 microbial species that collectively harbor more genes than the human genome itself [40]. This intricate microbial community serves as a cornerstone of human health, performing essential physiological functions that extend far beyond simple digestion and influence multiple organ systems throughout the body. The GM exhibits remarkable taxonomic diversity while maintaining consistent patterns across healthy individuals. Bacteria constitute the predominant and most extensively studied component, with the normal human GM primarily dominated by two major phyla: *Bacteroidetes *and *Firmicutes*, which together account for over 90% of the gut bacterial population [40, 41]. The remaining microbial community consists of *Actinobacteria*, *Proteobacteria*, *Fusobacteria*, and *Verrucomicrobia*, which are present as frequent but minor constituents [40, 41]. Within these major phyla, specific genera demonstrate particular functional significance. The Firmicutes phylum encompasses more than 200 different genera, including *Lactobacillus*, *Bacillus*, *Clostridium*, *Enterococcus*, and *Ruminococcus*, with *Clostridium *genera representing 95% of the Firmicutes phylum [41]. The *Bacteroidetes *phylum consists of predominant genera such as *Bacteroides *and *Prevotella*, while the *Actinobacteria *include the *Bifidobacterium *genus [41]. Recent molecular analyses have revealed that the GIT harbors more than a billion bacterial cells, with each harboring over 1,000 species-level phylotypes [42]. This vast diversity is concentrated primarily at the species and strain levels, with studies identifying more than 5,600 bacterial spp. across individuals, while the number of unique genera remains limited to less than 130 [43]. This pattern indicates that GM diversity is most pronounced at the finest taxonomic levels, suggesting highly specialized ecological niches and functional roles. Beyond bacteria, the GM encompasses methanogenic archaea (*Methanobrevibacter smithii*), eukaryotes (primarily yeasts), and viruses (bacteriophages) [25]. These non-bacterial components, while numerically smaller, contribute significantly to the overall functional capacity and stability of the microbial ecosystem.

Metabolic functions and nutrient processing

The GM serves multiple essential physiological functions that are fundamental to human health. Primary among these is the fermentation of dietary components that resist digestion by host enzymes, particularly complex carbohydrates, dietary fibers, and resistant starches [44, 45]. This fermentative process represents a crucial metabolic partnership between host and microbe, converting otherwise indigestible substrates into bioactive compounds that support host physiology. SCFA production represents the most significant metabolic contribution of the GM. Through bacterial fermentation of complex carbohydrates, the microbiota generates primarily acetate, propionate, and butyrate in an approximate ratio of 3:1:1 [46, 47]. These SCFAs serve multiple critical functions: they provide energy substrates for colonic epithelial cells, with butyrate serving as the preferred fuel source for colonocytes; they help maintain gut barrier integrity; they regulate immune responses; and they modulate systemic metabolism through interactions with G-protein-coupled receptors [48-50]. The GM also demonstrates a remarkable capacity for amino acid fermentation, contributing to SCFA production through alternative pathways [51]. This process involves the degradation of dietary proteins and host-derived proteins, generating not only acetate and propionate but also branched-chain fatty acids such as isobutyrate and isovalerate [52]. The ability to metabolize both carbohydrate and protein substrates provides metabolic flexibility, ensuring continuous SCFA production even under varying dietary conditions.

The GM makes significant contributions to vitamin synthesis, particularly B-group vitamins and vitamin K, which are essential for numerous physiological processes [44, 45]. The microbial capacity for vitamin production has been recognized for over 40 years, with studies in germ-free animals demonstrating the critical importance of microbial vitamin synthesis for host health [45]. B-vitamin biosynthesis represents an important function, with gut bacteria capable of producing biotin, cobalamin (B12), folates, nicotinic acid, pantothenic acid, pyridoxine (B6), riboflavin (B2), and thiamine (B1) [45, 53]. Systematic genomic analysis of 256 common gut bacteria revealed that some bacterial genomes contain pathways for all eight B vitamins, while others contain none, indicating significant functional diversity within the microbial community [45]. The distribution of vitamin-producing capabilities varies significantly across bacterial phyla. *Bacteroidetes *appear to be the phylum with the greatest number of predicted B-vitamin producers, with over 90% of *Bacteroidetes *species (spp.) producing multiple B vitamins excluding B12 [45]. For riboflavin (B2) and biotin (B7), all microbes from the phyla *Bacteroidetes*, *Fusobacteria*, and *Proteobacteria *possess the necessary biosynthetic pathways [45]. Vitamin K synthesis represents another crucial microbial function, with germ-free animals developing hemorrhagic tendencies due to deficient vitamin K-dependent clotting factor synthesis [45]. Human studies have confirmed the physiological significance of microbially-produced vitamin K, with antibiotic treatment leading to decreased plasma prothrombin levels in individuals on low vitamin K diets [45]. Importantly, the GM demonstrates complex cross-feeding relationships in vitamin metabolism, with vitamin-producing bacteria supplying essential nutrients to non-producing species [45]. This metabolic interdependence contributes to ecosystem stability while potentially limiting vitamin availability to the host, as a significant proportion of microbially-produced vitamins may be consumed by other bacteria rather than absorbed by the host.

The GM plays a critical role in immune system development and regulation, with its influence extending from local mucosal immunity to systemic immune function [53-55]. This immunomodulatory function represents one of the most profound ways the microbiome influences human health, affecting both innate and adaptive immune responses. Early immune system development is fundamentally dependent on microbial colonization. The microbiota educates the developing immune system, promoting the differentiation of regulatory thymus (T) cells (Tregs) and T helper (h) type 17 (Th17) cells, thereby maintaining immune homeostasis [40, 54]. Studies using germ-free animals have revealed that proper immune system maturation requires the presence of commensal microflora, with certain developmental defects becoming irreversible if not corrected during critical early-life periods [56]. The gut microbiome influences multiple immune cell populations through direct and indirect mechanisms. Specific bacterial species can promote the differentiation of helper T cells into Th1, Th2, and Th17 subsets, or into Treg cells with anti-inflammatory properties [53, 55]. For example, *Clostridium *spp. can promote Tregs induction, while *Bacteroides fragilis *can signal Tregs to suppress pro-inflammatory Th17 responses [56]. Systemic immune modulation extends the GM influence beyond the gut, affecting immune cells in distant organs through circulating microbial metabolites and molecular signals [57, 58]. SCFAs, particularly butyrate, promote the differentiation of peripherally induced Tregs and can inhibit the development of systemic inflammation [58]. The GM also influences neutrophil migration and function, macrophage activation, and natural killer (NK) cell maturation [58].

The GM plays a fundamental role in maintaining intestinal barrier function through complex interactions with the mucus layer and epithelial cells [59-61]. The intestinal epithelium is covered with a dense mucus layer that serves as the first line of defense against microbial translocation, and this barrier system is both influenced by and influences the resident microbiota. Mucus composition and structure are significantly affected by microbial presence. The mucus layer consists primarily of extensively O-glycosylated mucins, particularly mucin 2 (MUC2) in the colon, which form an organized glycoprotein network [59, 62]. The GM can interact with mucin glycans both as adhesion receptors and as nutritional substrates, with certain bacterial species specializing in mucin degradation as a carbon and energy source [59, 63]. The GM contributes to mucus layer integrity through multiple mechanisms. Beneficial bacteria can stimulate mucin production and secretion, while specific bacterial metabolites help maintain the physical and chemical properties of the mucus barrier [60, 62]. SCFAs produced by commensal bacteria maintain the slightly acidic pH of the mucus layer, which creates unfavorable conditions for pathogenic bacteria while supporting beneficial microbes [64]. Barrier dysfunction can result from GM dysbiosis, leading to increased intestinal permeability and bacterial translocation. Studies have shown that mice lacking MUC2 mucin demonstrate increased susceptibility to bacterial infection and greater colonization by pathogenic species, highlighting the interdependence between microbial communities and barrier function [62].

The GM possesses extensive xenobiotic metabolizing capabilities that significantly influence drug bioavailability, efficacy, and toxicity [65-67]. This metabolic capacity represents a "second liver" within the human body, with microbial enzymes capable of performing diverse chemical transformations on pharmaceutical compounds and environmental chemicals. Microbial drug metabolism can lead to activation, inactivation, toxification, or altered stability of therapeutic compounds [66, 68]. Over 30 drugs have been identified as substrates for intestinal bacteria, with the GM performing reactions including reduction, hydrolysis, decarboxylation, dehalogenation, demethylation, and, importantly, conjugate hydrolysis reactions [50]. These transformations can dramatically alter the pharmacokinetic (PK) and pharmacodynamic (PD) properties of orally administered medications. Inter-individual variations in drug response often correlate with differences in GM composition and metabolic capacity [65, 69]. Geographic and age-specific patterns in xenobiotic-metabolizing enzyme abundance have been identified across different populations, suggesting that personalized medicine approaches may need to consider individual microbiome profiles [69]. The relationship between drug consumption patterns and the abundance of xenobiotic-metabolizing bacteria suggests adaptive responses within microbial communities [69]. Bidirectional interactions occur between drugs and the GM, with therapeutic compounds potentially altering microbial community composition and function, which in turn affects the metabolism of subsequently administered drugs [66, 68]. This dynamic relationship highlights the complexity of drug-microbiome interactions and their potential impact on therapeutic outcomes.

**Figure 3:**
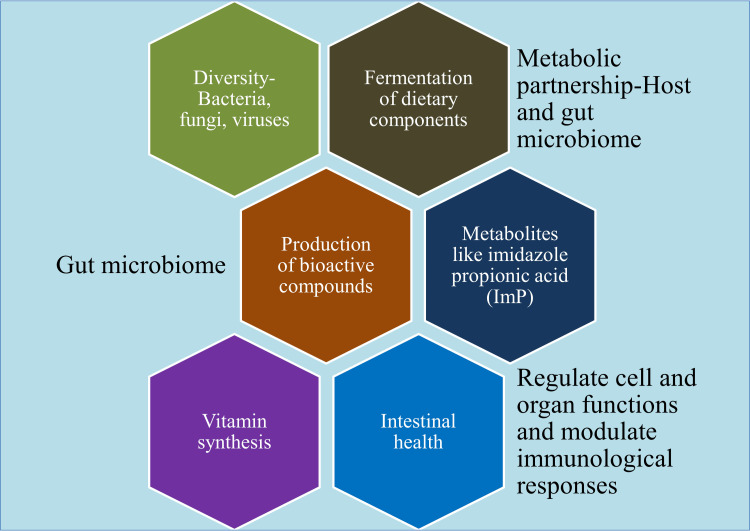
Functions of gut microbiome This image has been synthesized from references [17, 25, 40-69]. ImP: imidazole propionic acid.

The GM's composition and fundamental functions in human health represent a sophisticated biological system that has co-evolved with humans. The remarkable taxonomic diversity concentrated at the species and strain levels reflects highly specialized ecological niches and functional roles that collectively support host physiology. From essential metabolic functions, including SCFA production and vitamin synthesis, to immune system development and regulation, barrier function maintenance, and xenobiotic metabolism, the GM serves as an indispensable partner in human health. Understanding these fundamental functions provides the conceptual framework for investigating how specific microbial metabolites, such as ImP, may influence CV health through the complex interplay between GM and host physiology. The recognition that the GM functions as an integral component of human biology, rather than simply a collection of commensal organisms, has profound implications for our understanding of health, disease, and therapeutic interventions (Figure 3).

Gut microbiome dysbiosis and cardiovascular disease

GM dysbiosis, the disruption of the balanced microbial ecosystem within the intestine, has emerged as a pivotal driver of CV pathology. A diverse community dominated by *Firmicutes *and *Bacteroidetes *performs vital functions: fermentation of dietary fiber into SCFAs, synthesis of vitamins, maintenance of the mucosal barrier, and modulation of immune homeostasis. Dysbiosis manifests as reduced microbial diversity, loss of obligate anaerobes (e.g., *Faecalibacterium prausnitzii*), and overgrowth of pathobionts (e.g., *Escherichia coli*, *Klebsiella pneumoniae*), which compromises these functions and precipitates CV risk [70]. One key consequence of dysbiosis is increased intestinal permeability, also known as “leaky gut.” The reduced production of butyrate, the primary energy source for colonocytes, weakens tight junction integrity, allowing translocation of bacterial lipopolysaccharide (LPS) into the systemic circulation. Circulating LPS binds Toll-like receptor 4 (TLR4) on endothelial and immune cells, triggering Nuclear factor kappa-light-chain-enhancer of activated B cells (NF-κB)-mediated release of Interleukin (IL)-6, tumor necrosis factor alpha (TNF-α), and C-reactive protein (CRP). This chronic, low-grade endotoxemia accelerates endothelial dysfunction, promotes monocyte adhesion, and amplifies atherosclerotic plaque formation [71].

Dysbiosis also disrupts lipid metabolism through altered bile acid transformation and cholesterol handling. Under a balanced GM, also known as eubiome, microbial bile salt hydrolases convert primary bile acids into secondary bile acids that facilitate reverse cholesterol transport. In dysbiosis, shifts in bile acid pools favor a hydrophobic pro-inflammatory state, impairing cholesterol excretion and promoting lipid accumulation within arterial walls [71]. Concurrently, reduced SCFA production diminishes engagement of G protein-coupled receptor 41 (GPR41) and GPR43 receptors on VSMC and immune cells, impairing vasodilation and fostering hypertension [71]. The most extensively studied proatherogenic metabolite is TMAO. Dysbiotic communities enriched in choline trimethylamine (TMA) lyase (CutC)/CutC activating enzyme (CutD)-expressing bacteria convert dietary choline and L-carnitine into TMA, which hepatic flavin-containing dimethylaniline monoxygenase 3 (FMO3) oxidizes into TMAO. Elevated plasma TMAO correlates with increased risk of MI, stroke, and all-cause mortality, in part by enhancing foam cell formation, impairing reverse cholesterol transport, and augmenting platelet hyperreactivity [70, 71]. Each 10 µmol/L increment in TMAO elevates CV risk by approximately 7.6% [71]. Beyond metabolic derangements, dysbiosis skews immune cell balance toward pro-inflammatory phenotypes. Loss of SCFA-producing bacteria diminishes induction of Tregs, while expansion of pathobionts stimulates Th17 responses and inflammasome activation (e.g., nucleotide-binding oligomerization domain (NOD)-like receptor (NLR) pyrin containing 3 (NLRP3) protein), further amplifying vascular inflammation [71]. Detection of microbial DNA, such as *Streptococcus *and *Veillonella *spp., within human atherosclerotic plaques underscores the direct translocation of GM into vascular lesions, contributing to plaque instability [70].

**Figure 4:**
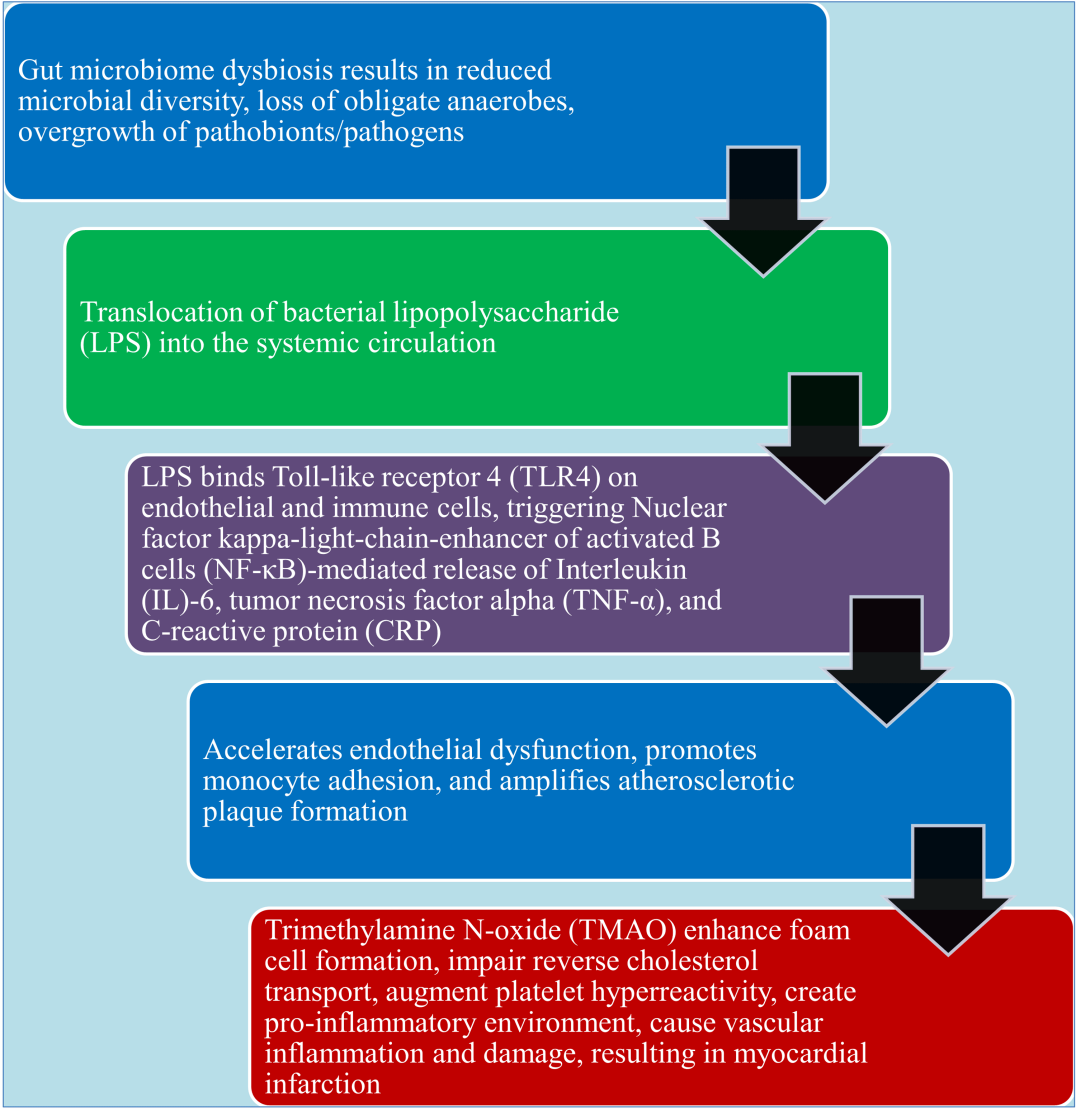
The process of gut microbiome dysbiosis resulting in cardiovascular complications This image has been synthesized from references [70, 71]. LPS: lipopolysaccharide; TLR4: toll-like receptor 4; NF-κB: nuclear factor kappa-light-chain-enhancer of activated B cells; IL-6: Interleukin-6, TNF-α: tumor necrosis factor alpha; CRP: C-reactive protein; TMAO: trimethylamine N-oxide.

Therapeutic strategies targeting dysbiosis aim to restore microbial equilibrium and mitigate CV risk. Probiotics (e.g., *Lactobacillus reuteri *National Collection of Industrial, Food and Marine Bacteria (NCIMB) 30242) and prebiotics (e.g., inulin) enhance SCFA production, strengthen barrier integrity, and lower LDL cholesterol and CRP levels [71]. Dietary interventions, notably the Mediterranean diet, rich in fiber, polyphenols, and unsaturated fats, promote beneficial taxa such as *Faecalibacterium prausnitzii*, increase SCFA output, and reduce TMAO generation, collectively decreasing major CV adverse events by ~30% in randomized trials [70]. Emerging approaches include FMT to reconstitute a balanced GM in metabolic syndrome, and preliminary studies demonstrate improvements in insulin sensitivity and lipid profiles post-FMT [70]. Pharmacological inhibitors of microbial TMA lyases (e.g., 3,3-dimethyl-1-butanol) show promise in preclinical models by reducing TMAO synthesis and attenuating atherosclerosis progression without broadly disrupting microbial diversity [71]. GM dysbiosis disrupts barrier function, skews immunometabolism, and drives production of proatherogenic metabolites-central mechanisms in CVD pathogenesis. Restoring eubiosis through dietary, microbial, and pharmacologic interventions offers a transformative avenue for CVD prevention and therapy. The continued integration of GM profiling into precision medicine will enable tailored strategies to correct dysbiosis and improve CV outcomes (Figure 4).

Imidazole propionic acid: sources and mechanisms

ImP is a GM-derived metabolite of the essential amino acid histidine that emerged as a critical mediator linking microbial metabolism to ATS and broader CV risk. Although histidine is obtained exclusively from dietary proteins-abundant in meat, fish, eggs, dairy, legumes, nuts, and whole grains-circulating ImP levels do not correlate directly with dietary histidine intake but rather reflect the abundance and activity of specific bacterial taxa harboring the UrdA enzyme in the GM [22, 72]. The initial step of microbial histidine catabolism involves histidine ammonia‐lyase (hutH), which deaminates histidine to trans‐urocanate; trans‐urocanate undergoes isomerization to cis‐urocanate and is then reduced by UrdA to yield ImP, a reaction that is pH‐sensitive and optimized under neutral colonic conditions prevalent in dysbiosis [22, 73]. UrdA homologs have been identified in diverse gut bacteria, including *Streptococcus mutans*, *Eggerthella lenta*, *Clostridium symbiosum*, *Ruminococcus gnavus*, *Veillonella *spp., and certain *Lactobacillus *spp., and their prevalence is enriched in individuals consuming a diet high in saturated fats and low in fiber [22, 74].

Following microbial synthesis in the colon, ImP is absorbed into the portal circulation and distributed systemically, where elevated levels exert pleiotropic effects on vascular and metabolic homeostasis. In endothelial cells, ImP impairs insulin receptor signaling by activating the Mitogen-Activated Protein Kinase (MAPK)-p38γ-Sequestosome-1 (p62)-Mechanistic Target of Rapamycin Complex 1 (mTORC1) pathway, leading to reduced Insulin Receptor Substrate 1 (IRS‐1/2) phosphorylation, diminished PKB activation, and sustained nuclear retention of FOXO1, culminating in decreased NO production, impaired vasodilation, and compromised endothelial repair after injury [23]. This endothelial dysfunction translates to increased monocyte adherence and vascular inflammation, as ImP upregulates ICAM‐1, VCAM‐1, and E-selectin, and correlates clinically with elevated soluble VCAM‐1 and CRP levels in patients with CAD [23, 25]. Animal studies in Apoe⁻/⁻ mice demonstrate that chronic ImP exposure increases atherosclerotic lesion area by approximately 25%, independent of plasma cholesterol, with plaques exhibiting enhanced smooth muscle activation and macrophage infiltration [23]. Beyond direct vascular effects, ImP disrupts systemic metabolic regulation. In hepatocytes, ImP activation of p38γ-p62-mTORC1 induces insulin resistance and attenuates metformin efficacy by inhibiting Adenosine Monophosphate-activated Protein Kinase (AMPK), contributing to dysglycemia and chronic inflammation [24, 72]. Clinical cohorts corroborate that higher ImP associates with increased Homeostatic Model Assessment of Insulin Resistance (HOMA‐IR) and glycated hemoglobin, independent of BMI and histidine intake [29]. Furthermore, HF patients exhibit elevated ImP levels that predict reduced ejection fraction and five‐year mortality, underscoring ImP’s relevance across the spectrum of CVD severity [26, 75].

The metabolic itinerary of ImP encompasses dietary histidine catabolism, microbial conversion, host absorption, signaling interactions, and excretion. After UrdA‐mediated reduction, ImP competes with protective SCFAs for GPR41 and GPR43 receptors on vascular and immune cells, shifting signaling toward pro‐inflammatory and vasoconstrictive states [23, 24]. Concurrent dysbiosis also elevates other proatherogenic metabolites such as TMAO and hydrophobic bile acids, compounding lipid dysregulation and endothelial injury [25]. Impaired renal excretion in CKD further augments systemic ImP burden, linking ImP to comorbid CV-renal pathologies. Importantly, diet and GM interventions can modulate ImP production. High‐fiber, unsaturated fat-rich diets suppress UrdA‐carrying bacteria and lower ImP, whereas Western diets enhance ImP‐producers [74]. Probiotic and prebiotic strategies that restore SCFA producers and acidify the colon may reduce UrdA activity, decreasing ImP synthesis [24]. Emerging pharmacological inhibitors targeting UrdA’s active site, informed by high‐resolution crystal structures, hold promise for directly blocking ImP production without broadly disrupting microbial diversity [76]. FMT and ELMs with inactivated UrdA offer additional precision approaches under investigation [77]. ImP represents a microbiome‐derived metabolite that integrates dietary inputs, microbial ecology, and host signaling to drive atherosclerosis and CV risk through endothelial dysfunction, inflammation, and metabolic derangements. Its production is highly responsive to diet and microbial composition, making ImP a dynamic biomarker and therapeutic target in the gut-heart axis. Future research should prioritize longitudinal profiling of ImP during plaque development, causal mediation analyses disentangling ImP from broader dysbiosis, and clinical trials of UrdA inhibitors or microbiome‐targeted interventions to mitigate ImP’s vascular and metabolic effects.

Imidazole propionic acid in atherosclerosis and cardiovascular risk

ImP is a histidine‐derived microbial metabolite that has emerged as a mediator in the pathogenesis of ATS and broader CVD. Although histidine intake provides the substrate for ImP synthesis, circulating ImP levels correlate more closely with the presence and activity of specific gut bacteria than with dietary histidine itself. Through its pleiotropic effects on endothelial function, inflammation, lipid metabolism, and insulin signaling, ImP integrates GM dysbiosis with vascular pathology, offering both mechanistic insights and potential therapeutic targets in CV medicine. Central to ImP’s pro‐atherogenic action is its capacity to impair endothelial repair and nitric oxide-mediated vasodilation. In human aortic endothelial cells, ImP dose-dependently inhibits PI3K/PKP signaling downstream of the insulin receptor, resulting in sustained activation of the FOXO1 transcription factor and reduced endothelial migration and angiogenesis. These in vitro findings translate in vivo, where chronic ImP administration compromises carotid artery re-endothelialization after injury and abrogates acetylcholine‐induced, endothelium‐dependent relaxation of murine aortae [23]. Such endothelial dysfunction constitutes an early and critical event in atherogenesis, fostering monocyte adhesion and ameliorating vascular resilience. Indeed, proinflammatory activation of the endothelium underlies several of ImP’s vascular effects. ImP upregulates adhesion molecules, including ICAM-1, VCAM-1, and E-selectin on endothelial surfaces, enhancing monocyte recruitment into the intima and amplifying local inflammation. Elevated circulating ImP correlates with higher levels of soluble VCAM-1 and CRP in patients undergoing cardiac evaluation, linking dysbiosis‐driven metabolite production to systemic endothelial activation [23]. The causal role of ImP in plaque development is further supported by animal models. In Apoe⁻/⁻ mice fed a high‐fat diet, daily exposure to ImP (800 µg/day) for 12 weeks increased aortic atherosclerotic lesion area by approximately 25% without affecting plasma cholesterol levels. Lesions in ImP‐treated mice exhibit enhanced smooth muscle α‐actin expression and greater TCD68⁺ macrophage infiltration, demonstrating lipid‐independent acceleration of plaque progression [23]. Beyond direct vascular injury, ImP contributes to metabolic dysregulation that amplifies CV risk. ImP impairs insulin signaling in hepatocytes via the p38γ-p62-mTORC1 axis, inducing insulin resistance and reducing the glycemic efficacy of metformin. In clinical cohorts, higher plasma ImP levels are associated with increased HOMA-IR and glycated hemoglobin independent of histidine intake and body mass index, thus exacerbating dysglycemia potent driver of atherogenesis [22]. Observational studies in humans confirm the clinical relevance of ImP in CVD. In a cohort of 831 patients undergoing elective cardiac angiography, individuals in the highest quartile of plasma ImP had a four‐fold increased odds of significant coronary artery disease even after adjustment for traditional risk factors, including hypertension, dyslipidemia, DM, and smoking [adjusted odds ratio (OR) 4.22; 95% confidence interval (CI), 2.60-6.97] [23]. Similarly, in people with HIV infection population at elevated CV risk-circulating ImP levels were significantly higher in those with obstructive CAD and correlated with gut microbial dysbiosis featuring enrichment of *Ruminococcus gnavus *and *Veillonella *spp., known ImP producers [25]. ImP’s impact extends to HF, where elevated serum ImP independently predicts reduced left ventricular ejection fraction and five‐year mortality. In two large cohorts (European, n=1,985; North American, n=2,155), patients in the highest ImP quartile exhibited an 85% increased risk of death over five years [adjusted hazard ratio (HR) 1.85; 95% CI, 1.20-2.88], underscoring ImP’s prognostic significance across CV phenotypes [26].

**Figure 5:**
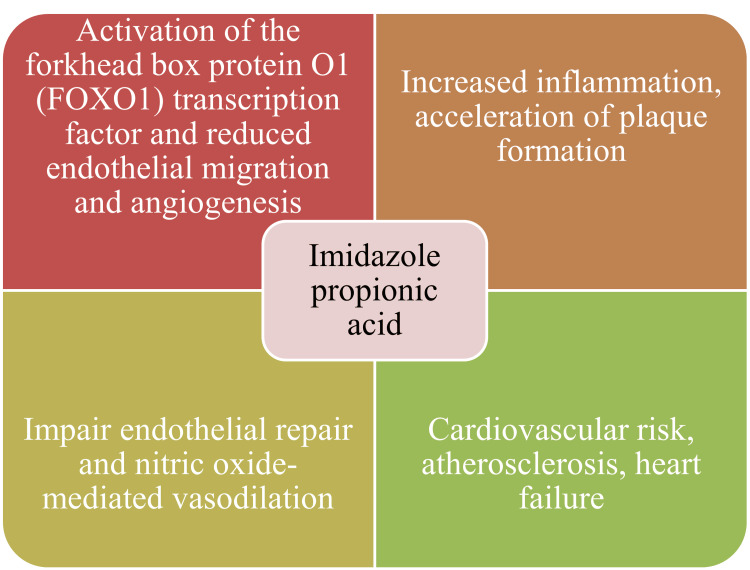
The role played by imidazole propionic acid in the development of cardiovascular complications This image has been synthesized from references [22-26]. FOX01: forkhead box protein O1.

Mechanistically, ImP competes with protective SCFAs for GPR41 and GPR43 on vascular and immune cells, signaling proinflammatory and vasoconstrictive pathways. Concurrent dysbiosis often elevates other proatherogenic metabolites, including trimethylamine-N-oxide and hydrophobic secondary bile acids, compounding lipid dysregulation and endothelial injury [22]. The microbial origins of ImP lie in the urocanate pathway. Specific gut bacteria possessing hutH and UrdA convert dietary histidine to ImP. UrdA homologs have been identified in taxa such as *Streptococcus mutans*, *Eggerthella lenta*, *Clostridium symbiosum*, *Ruminococcus *gnavus, and *Veillonella*. Western diets rich in saturated fat and low in fiber foster these ImP producers, raising systemic ImP levels, whereas high-fiber, unsaturated-fat diets suppress UrdA‐carrying bacteria and lower ImP synthesis [22, 23]. Recognizing ImP’s role in ATS opens avenues for microbiome‐targeted therapies. Strategies include dietary modulation to enrich SCFA producers and acidify colonic pH, probiotics or prebiotics to restore eubiosis, and direct inhibition of UrdA. High-resolution crystal structures of UrdA provide templates for small-molecule inhibitors aimed at blocking ImP production without broad GM disruption. Early studies of pirfenidone, p38γ antagonist-and ginsenoside compound K demonstrate proof‐of-concept for mitigating ImP-mediated signaling in metabolic disease models [24]. ImP represents a gut‐derived metabolic link between dysbiosis and CV pathology, acting through endothelial dysfunction, inflammation, and metabolic derangements to promote ATS and HF. Its production is modifiable via diet, microbiome interventions, and enzymatic inhibitors, positioning ImP as both a biomarker and therapeutic target in precision CV medicine. Future research should focus on longitudinal profiling of ImP during plaque development, causal mediation analyses disentangling ImP from broader dysbiosis, and interventional trials of UrdA inhibitors or GM‐targeted strategies to reduce ImP and improve vascular outcomes (Figure 5).

Future directions, research gaps, and therapeutic potential

Elucidating ImP as a driver of ATS has opened avenues for advancing CV precision medicine. To translate mechanistic insights into clinical usefulness, concerted efforts must address foundational gaps in ImP biology, improve risk stratification, and develop targeted interventions spanning diet, microbiome modulation, and pharmacology.

Unresolved mechanistic questions

Despite clarity on the UrdA pathway-hutH converting histidine to urocanate and UrdA reducing urocanate to ImP, ecological and molecular determinants of ImP synthesis remain incompletely defined. Strain-level variation within UrdA-harboring taxa (e.g., *Ruminococcus gnavus*, *Eggerthella lenta*, *Streptococcus mutans*) influences net ImP production and requires systematic metagenomic and enzymatic profiling across diverse human populations [22]. The impact of colonic luminal pH, shaped by dietary fiber fermentation, on UrdA activity and ImP yield warrants investigation using gnotobiotic mouse models and controlled feeding trials [23]. Additionally, host mechanisms of ImP clearance via renal excretion and hepatic metabolism are unexplored; understanding transporter-mediated uptake and potential metabolic conjugation is essential, particularly for patients with CKD, where impaired clearance may amplify vascular injury.

Longitudinal and population-based cohorts

Most clinical data on ImP and CV phenotypes derive from cross-sectional angiography or HF cohorts, limiting causal inference. Prospective studies integrating serial plasma ImP measurements with noninvasive imaging (carotid ultrasound, coronary calcium scoring) can elucidate temporal relationships between ImP elevations, plaque progression, and clinical events [22, 23]. The inclusion of ethnically diverse and underrepresented populations is critical, given the GM variation by geography and diet. The Coronary Artery Risk Development in Young Adults (CARDIA) and Health Literacy Instrument for Adults (HELIUS) cohorts provide frameworks for embedding ImP assays in longitudinal CV research [22]. Life-course analyses-tracking ImP from young adulthood through senescence-will clarify whether ImP contributes primarily to plaque initiation, progression, or rupture.

Dietary and lifestyle interventions

Dietary strategies represent immediately translatable means to modulate ImP. High-fiber, unsaturated-fat diets shift GM toward SCFA producers, lowering colonic pH and suppressing UrdA‐carrying bacteria, thus reducing ImP synthesis in humans and rodents [22, 23]. Randomized feeding trials comparing Mediterranean, plant-based, and Western dietary patterns, with ImP as a pre-specified biomarker, can establish efficacy and inform dietary guidelines for CV risk reduction. Lifestyle factors, like physical activity, stress, and avoidance of broad-spectrum antibiotics, should be examined for their impact on ImP dynamics and CV endpoints.

Microbiome-targeted therapies

Probiotics, prebiotics, and FMT offer avenues to reshape gut ecology and diminish ImP producers. Rationally engineered probiotics lacking UrdA or prebiotics that preferentially nourish SCFA producers require testing for safety, colonization, and ImP reduction in humans [77]. Preliminary FMT studies in metabolic syndrome demonstrate modulation of circulating microbial metabolites; embedding ImP measurements in these trials will determine whether microbiome reconstitution translates to lower ImP and improved endothelial function.

Pharmacological inhibitors of ImP synthesis and signaling

High-resolution crystal structures of bacterial UrdA present templates for rational drug design. Small-molecule inhibitors targeting UrdA’s flavin adenine dinucleotide (FAD)-binding pocket have been proposed, with early candidates achieving submicromolar potency in vitro [22, 78]. Preclinical studies must assess PK, off-target effects, and GM impact. Parallel efforts should explore inhibitors of histidine ammonia-lyase (hutH) to reduce upstream substrate availability. On the host side, antagonists of ImP-activated pathways-p38γ MAPK, mTORC1, and ImP’s putative imidazoline receptor 1- represent additional therapeutic angles [22]. Pirfenidone analogs, which compete with ImP for p38γ binding, restored insulin signaling in preclinical models and merit advancement to phase I clinical trials to evaluate safety and biomarker modulation in humans.

Biomarker development and clinical integration

To establish ImP as a robust biomarker, assay standardization across laboratories using the ultra-high performance liquid chromatography-mass spectrometry (HPLC-MS) is essential. Defining normal and pathological reference ranges in healthy and at-risk cohorts will enable risk stratification. Integrating ImP into established CV risk scores may enhance the prediction of incident events in intermediate-risk persons. In therapeutic trials, demonstrating that ImP reduction correlates with improved endothelial function, plaque regression, or fewer CV events will validate ImP as a surrogate endpoint.

Systems biology and precision medicine

A multidisciplinary integration of microbial metagenomics (the study of genetic material), metabolomics (the study of metabolites), transcriptomics (the study of ribonucleic acid molecules), and host genomics will elucidate ImP’s network effects and identify modifiers of its pathogenicity as host receptor polymorphisms or comorbid microbiome alterations. Machine-learning (ML) approaches can combine microbial UrdA capacity, circulating ImP levels, dietary patterns, and genetic risk to personalize preventive and therapeutic plans. Collaborative consortia, mirroring efforts like MetaCardis, an European Union (EU)-funded project investigating the role of GM in the development of cardiometabolic diseases, but focused on ImP and CV outcomes, will accelerate data sharing, harmonize protocols, and foster innovation.

## Conclusions

The emerging body of evidence positions ImP as a pivotal mediator bridging GM dysbiosis and CVD. Through a multi‐faceted interplay of endothelial dysfunction, pro‐inflammatory signaling, and metabolic derangements, ImP contributes to each stage of atherogenesis, endothelial activation, and monocyte adhesion to plaque progression and instability. Unlike traditional risk factors, ImP reflects the dynamic state of the GM and its metabolic output, offering a window into residual CV risk that persists despite optimal management of lipids, blood pressure, and glycemia. Crucially, the biosynthesis of ImP via the UrdA pathway underscores the importance of microbial ecology. Thus, ImP constitutes both a biomarker of gut‐heart axis perturbation and a mechanistic link to vascular injury. Translational research to date has delineated key nodes for intervention: dietary modulation to reshape colonic pH and microbial composition; probiotics, prebiotics, and FMT to restore eubiosis; and targeted pharmacologic agents such as UrdA inhibitors and p38γ antagonists to block ImP synthesis and signaling. Early animal and in vitro studies validate ImP’s direct impact on endothelial insulin signaling, NO bioavailability, and monocyte recruitment, while human cohorts consistently associate elevated ImP with higher odds of CAD, greater plaque burden, diminished left ventricular function, and increased mortality in HF. These findings not only affirm ImP’s pathogenic role but also highlight its potential as a surrogate endpoint in clinical trials. Nevertheless, critical knowledge gaps remain. The relative contributions of individual UrdA‐expressing microbial species to total ImP production, the modulation of UrdA activity by colonic microenvironmental factors, and the host’s capacity for ImP clearance require deeper investigation. Longitudinal studies in diverse populations are needed to establish causal relationships and temporal trajectories of ImP in the plaque initiation, progression, and regression. Moreover, standardization of ImP quantification methods and establishment of normative reference ranges will be foundational for integrating ImP into risk stratification algorithms and therapeutic monitoring.

Looking forward, the integration of ImP profiling into precision CV medicine holds promise for identifying individuals with microbiome‐driven residual risk. By combining ImP levels with genomic, metabolomic, and imaging data, clinicians can develop personalized interventions, ranging from dietary prescriptions and GM‐modifying therapies to novel small‐molecule inhibitors that specifically target gut‐derived atherogenic mechanisms. Collaborative, multidisciplinary consortia will be essential to validate ImP as both a biomarker and a treatment target, ensuring that microbiome research translates into tangible improvements in CV outcomes. In summary, ImP represents a novel axis of CV risk that transcends traditional paradigms by embedding microbial metabolism at the core of atherogenesis. Its dynamic nature, modifiability, and mechanistic potency render ImP a compelling target for future research and therapeutic development. Addressing the remaining mechanistic and translational challenges will not only advance our understanding of the gut-heart axis but also open new avenues to reduce the global burden of ASCVD.
